# A Mendelian Randomization Study and Experimental Validation Investigating the Potential Relationship Among Interleukin‐6 Receptor Subunit Beta, Obesity, and Alzheimer's Disease

**DOI:** 10.1002/brb3.70772

**Published:** 2025-09-16

**Authors:** Yu Liu, Nan Song, Qun Wang, Peng Cui, Dongyu Min

**Affiliations:** ^1^ Liaoning University of Chinese Medicine Shenyang Liaoning China; ^2^ Affiliated Hospital of Liaoning University of Traditional Chinese Medicine Shenyang Liaoning China

**Keywords:** Alzheimer's disease, interleukin‐6 receptor subunit beta, Mendelian randomization, obesity

## Abstract

**Objective:**

This study employs Mendelian randomization (MR) aimed at systematically evaluating the relationship among interleukin‐6 receptor subunit beta, obesity, and Alzheimer's disease (AD). We conducted animal studies to validate the reliability of the MR analytical outcomes.

**Methods:**

The pooled data for the interleukin‐6 receptor subunit beta originated from the genome‐wide association study (GWAS) dataset, which included a total of 10,534,735 participants. Obesity pooled data were from the GWAS dataset (case *n* = 23,971 and control *n* = 388,084) and AD pooled data from the GWAS database (case *n* = 39,106 and control *n* = 46,828). The aforementioned data sets facilitated MR causal analysis. First, utilize the inverse variance weighting (IVW) method for analysis and enhance it with MR‐Egger regression and weighted median approaches, and a sensitivity analysis was performed by MR‐multiple effect residuals and outliers (MR‐Presso), Cochran Q test, and Leave‐one (LOO) analysis. We established an obesity model by feeding 6‐week‐old male ApoE^−/−^ mice a high‐fat diet for 16 weeks. In contrast, C57BL/6 control mice were fed a normal diet for the same duration. An AD model was established by feeding 3‐month‐old APP/PS1 mice a normal diet for 24 weeks. We harvested serum and hippocampal tissue from the mice for enzyme‐linked immunosorbent assay (ELISA).

**Results:**

MR analysis indicated that a genetically predicted increase in interleukin‐6 receptor subunit beta raises the risk of AD (OR = 1.064, 95% CI: 1.021–1.109, *p* = 0.003). The exposure factor interleukin‐6 receptor subunit beta served as a protective element against obesity (OR = 0.9372,95%CI:0.8921–0.9847, *p* = 0.010). Obesity showed an adverse relationship with AD. As the body mass index (BMI) increased, the risk of developing AD decreased (OR = 0.9299, 95% CI: 0.8939–0.9674, *p*  ＜0.001). ELISA findings revealed that the levels of interleukin‐6 receptor subunit beta (gp130), oncostatin‐M (OSM), and IL‐6 in serum and hippocampus decreased in obesity, whereas they increased in AD, aligning with the results of the MR Analysis.

**Conclusion:**

In summary, our extensive Mendelian randomization data suggest that increased levels of the interleukin‐6 receptor subunit beta may be associated with a reduced risk of obesity, and consequently, may increase the risk of AD.

## Graphical abstract

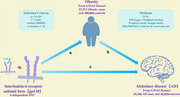

## 1 Introduction

Alzheimer's disease (AD) is an incurable, diverse neurodegenerative disorder. It features short‐term memory loss, compromised judgment, and difficulties in problem‐solving. Additionally, it causes mood and behavioral shifts. The fundamental biology involves the buildup of extracellular beta‐amyloid plaques and the formation of neuronal tangles due to hyperphosphorylated tau protein within cells. It also involves neuroinflammation, dysfunction in synapses and circuits, mitochondrial disturbances, energy metabolism issues, epigenetic alterations, and vascular irregularities (Knopman et al. 2021; Cummings 2021; Pimenova et al. 2018). The increase in AD patients will bring a great economic burden to mankind. According to relevant statistics, there are about 50 million dementia patients in the world, of which AD accounts for about 2/3. In addition, as the population ages, Alzheimer's disease (AD) prevalence increases, potentially impacting 135 million individuals by 2050 (Khan et al. 2020). Thus, it is essential to explore the risk factors linked to AD and identify effective treatments to reduce both incidence and mortality rates, which will also improve patients' quality of life.

Interleukin‐6 receptor subunit beta, or glycoprotein 130 (gp130), serves as a signaling receptor within the IL‐6 cytokine family, which includes IL‐6, IL‐11, oncostatin M (OSM), cardiotrophic factor 1 (CT‐1), leukemia inhibitory factor (LIF), and ciliary neurotrophic factor (CNTF) (Hong et al. 2016; Miyaoka et al. 2006). The IL‐6 cytokine family works through a receptor complex containing at least one Interleukin‐6 receptor subunit beta, where IL‐6 and IL‐11 bind to the homodimer of Interleukin‐6 receptor subunit beta upon binding to specific receptors, and OSM binds to the heterodimer formed by OSM receptor and Interleukin‐6 receptor subunit beta (Peters et al. 1998). It has to be said that the interleukin‐6 receptor subunit beta is necessary for the IL‐6 cytokine family to function. Some recent studies have shown that IL‐6 cytokine family is closely related to AD, among which OSM secretion increases in patients with AD and decreases after treatment with acetylcholinesterase inhibitors (Reale et al. 2005), TSG treatment can inhibit the production of inflammatory cytokines such as IL‐6 and suppress neuroinflammation to prevent cognitive impairment in Alzheimer's disease (Gao et al. 2023). The above studies can indirectly indicate that there is a correlation between Interleukin‐6 receptor subunit beta and AD, but the relationship between the two is still unknown.

Obesity is a chronic condition characterized by excessive body fat accumulation. It is clinically identified using the body mass index (BMI), which classifies an individual as obese when their BMI exceeds 30 (Micioni Di Bonaventura et al. 2020). Observing data from the World Health Organization (WHO), it can be found that approximately 890 million adults worldwide will suffer from obesity in 2022, about 16% of adults aged 18 and older, and the global prevalence of obesity has more than doubled from 1990 to 2022. Previous studies have found that Interleukin‐6 receptor subunit beta is an important component of the IL‐6 family in ameliorating obesity, in which OSM, CNTF, IL‐6, and other cytokines can reduce adipogenesis via interleukin‐6 receptor subunit beta (White et al. 2008; Piquer‐Garcia et al. 2020; Timper et al. 2017). In addition, obese people are more likely than normal‐weight people to develop comorbidities such as cardiovascular disease, sleep apnea, type 2 diabetes, and AD (Wilson et al. 2017). Experts agree that obesity is a risk factor for AD (Crous‐Bou et al. 2017), but this may be specific to midlife obesity, because recent studies have found that as obesity ages, the risk of dementia is steadily decreasing (Wotton et al. 2014), obesity in old age may actually reduce the risk of AD (Osiecka et al. 2023). Middle‐aged obese patients will show an increase in beta amyloid precipitation in the hippocampus, which is similar to the pathological changes in AD patients, and bariatric surgery can improve this pathological change and prevent pathological cognitive decline and even the development of AD (Alosco et al. 2014; Chuang et al. 2016). Interestingly, obesity in older adults may reduce AD risk. A long‐term study spanning from 1968 to 32 years revealed that maintaining a higher BMI in older age helps to safeguard against cognitive decline and AD (Gustafson et al. 2009). In summary, it is reasonable to speculate that there may be a causal link among interleukin‐6 receptor subunit beta, obesity, and AD, and that choosing an approach to elucidate these associations may help to better intervene and manage patients with AD at an early stage and reduce the incidence of AD and its complications.

Mendelian randomization (MR) is a study design that uses instrumental variables to analyze and test causal hypotheses in non‐experimental data. It is conducted using existing large‐scale GWAS data, which can solve some issues and shortcomings of previous observational research methods and randomized controlled trials (Larsson et al. 2023).

Therefore, this research takes obesity and AD as the outcomes, gp130 and obesity as the exposures, uses MR to explore the correlation between interleukin‐6 receptor subunit beta, obesity, and AD, and then uses an Elisa experiment to verify it, in order to gain a new perspective on preventing and addressing obesity and AD.

## 2 Methods

### 2.1 Study Design and DRAWINGS

**FIGURE 1:**
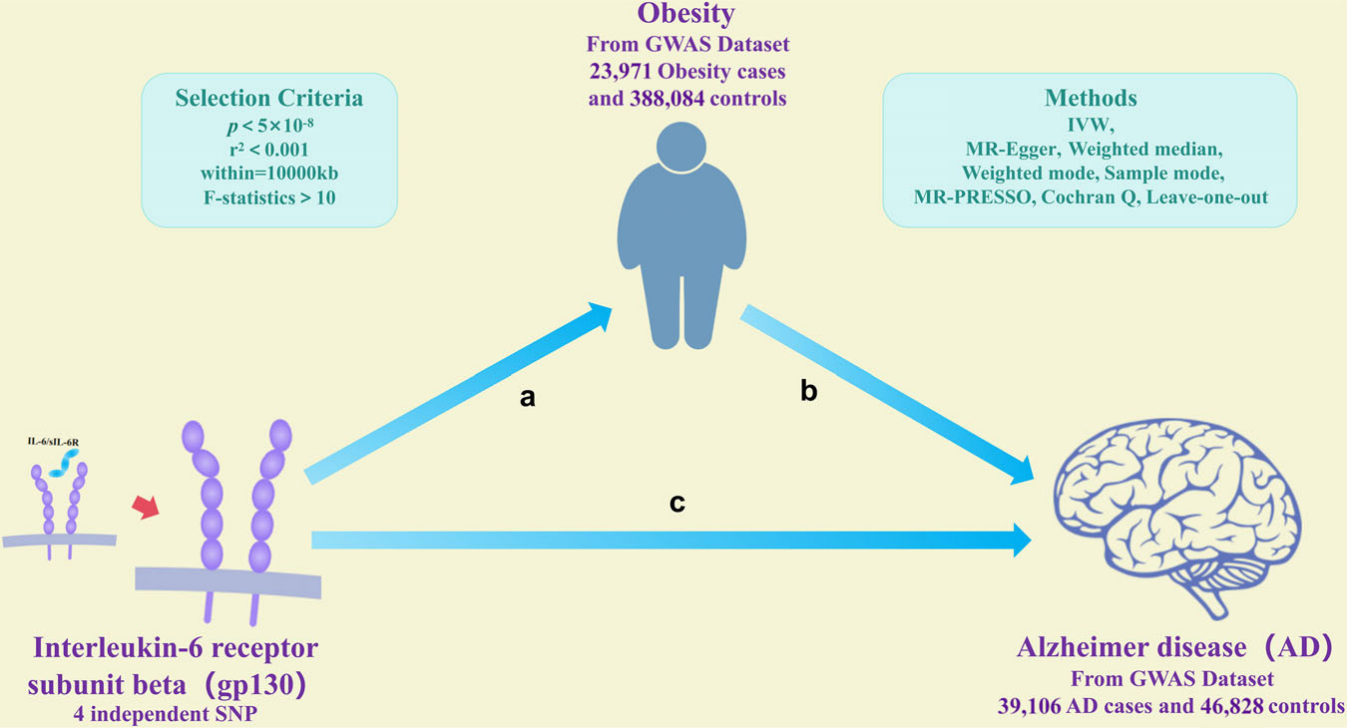
Conceptual diagram of two‐sample MR of causality proposed between interleukin‐6 receptor subunit beta, obesity, and AD.

Figure 1 shows the research design diagram of the study. This study used a two‐sample MR approach, using publicly available datasets, to elucidate the hypothesized causal connection among interleukin‐6 receptor subunit beta, obesity, and AD. Instrumental variables (IVs) must meet three conditions: (1) reliable correlation with exposure; (2) The results were correlated only by exposure; and (3) the results were independent of unobserved confounders (Davey et al. 2014). Therefore, in the present study, single‐nucleotide polymorphism (SNP) was selected as the IVs. On the basis of meeting the above conditions, nterleukin‐6 receptor subunit beta and obesity were identified as exposure factors, and obesity and AD were identified as outcome factors, to determine a causal connection between the three.

### 2.2 Data Sources

In this research, the data for the interleukin‐6 receptor subunit beta come from the IEU database (https://gwas.mrcieu.ac.uk/), which contains a total of 10,534,735 SNPs.The comprehensive GWAS summary data for obesity can be found in gs://finngen‐public‐data‐r10/summary_stats/finngen_R10_E4_OBESITY.gz, which includes 23,971 obesity cases and 388,084 controls. All the cohort comprised European individuals diagnosed with Obesity. From https://gwas.mrcieu.ac.uk/datasets/ebi‐a‐GCST90027158/ obtain GWAS data related to AD used in this study. This GWAS includes 39,106 cases of AD and 46,828 control cases, a cohort that included Europeans diagnosed with AD. A total of 20, 921,626 SNPs for AD exist in this database.

### 2.3 Genetic Tool Variable Selection

In our study, SNPs closely related to exposure were obtained in strict accordance with the selection criteria of IVs, satisfying the selection of genetic predictors *p* < 5×10^−8^, which can avoid the influence of existing interference factors in observational research. To minimize bias caused by linkage disequilibrium (LD) and preserve the independence of SNPs, we strictly applied an LD threshold *r* ^2^ < 0.001 in the range of 10 000 KB when grouping all SNPs, and excluded palindrome SNPs from the analysis. In addition, to mitigate instrumental variable bias, we calculate the *F* statistic for each SNP and select IV with *F* > 10, excluding variables with *F* less than 10 from our study.

### 2.4 Statistical Analysis of MR

#### 2.4.1 MR Analysis

In the present MR analysis, we used a two‐sample MR approach to assess the potential connection between the interleukin‐6 receptor subunit beta and AD. The analyses were dominated by the inverse variance weighting (IVW) method. Since all SNPs are considered valid, IVs are the basis of the IVW analysis method, so the IVW approach yields causal effect estimates with strong confidence. First, the effects of individual exposure factors on outcomes were assessed using the Wald ratios, which were then combined to produce estimates of primary causality. When heterogeneity was *p* < 0.05, a random effects model was used (Yu et al. 2024). Otherwise, the fixed effects model is used. In addition, we supplement and validate IVW using MR‐Egger regression and weighted median methods. The weighted median method is slightly less accurate than IVW, but more accurate than MR‐Egger, which stipulates that more than half of the variables in the analysis must come from valid instruments. MR‐Egger is a method to detect possible polysemy in genetic tools, and when all tools are ineffective, it can adjust the results accordingly and provide a more accurate estimate of correction (Li et al. 2024).

#### 2.4.2 Sensitivity and Heterogeneity Analysis

Sensitivity analysis of MR analysis can evaluate its reliability, usually using MR‐multiple effect residuals and outliers (MR‐PRESSO), Cochran Q test, and leave‐one‐out(LOO) analysis for sensitivity analysis. The MR‐PRESSO method is used to detect the potential existence of horizontal pleiotropy. MR‐PRESSO detects possible outliers and removes them to produce a relatively unbiased causal estimate. The Cochran Q test is used to detect whether there are significant differences among multiple dependent variables in a single group. If *p* < 0.05 of the Cochran Q test, heterogeneity is considered. LOO is used to evaluate the impact of observations on sample data after excluding the overall results.

The application of R software (version 4.2.1) was chosen for all statistical analyses. For causality estimation, we chose to apply the “Two Sample MR” software package (version 0.5.6), and the “MR‐PRESSO” package was used to detect the presence of outliers. FDR q values were estimated using the R package “p.adjust.”

### 2.5 Experimental Validation of MR Analysis Results

#### 2.5.1 Animals

Six‐week‐old male ApoE^−/−^ obesity mice, C57BL/6 control mice, and 3‐month‐old male APP/PS1 AD mice were housed at 22°C with a 12‐h light/dark cycle. The mice had free access to food and water. All experiments were approved by the Animal Research Ethics Committee of Liaoning University of Traditional Chinese Medicine and the Animal Ethics Committee of the Affiliated Hospital of Liaoning University of Traditional Chinese Medicine and were conducted in accordance with the national regulations on animal experimentation in China.

#### 2.5.2 In Vivo Studies

C57BL/6 mice served as the control group, ApoE^−/−^ mice as the obesity group, and APP/PS1 mice as the AD group. To establish the obesity model, the obesity group was fed a high‐fat diet for 16 weeks (Yao et al. 2023), while the control group was fed a normal diet during the same period. The Alzheimer's disease group was fed a normal diet for 24 weeks. After euthanasia, blood samples and hippocampal tissue were collected from the mice and stored at −80°C for subsequent enzyme‐linked immunosorbent assay (ELISA).

#### 2.5.3 ELISA

Elisa kits were employed to detect the levels of gp130, IL‐6, and OSM; followed the kit instructions. Mice serum, and hippocampal tissue frozen at −80°C were taken, and the hippocampal tissue was ground into appropriate normal saline to prepare tissue homogenate. Standard holes, sample holes, and blank holes were set according to the ELISA kit instructions. Standard holes were added with 50 µL standard product of different concentrations, sample holes were added with 10 µL sample and 40 µL sample diluent, and 100 µL horseradish peroxidase (HRP)‐labeled detection antibody was added to the sample holes, incubated at 37°C for 1 h, and then discarded and patted dry. The washing solution was added to each hole, let to sit for 1 min, and then discarded the washing solution and repeated washing five times. Then, 50 µL of substrate A and substrate B were added to each well, and 50 µL of termination solution was added after incubation at 37°C for 15 min without light. The optical density (OD) of each hole was measured at a wavelength of 450 nm, and the results were recorded using an excel spreadsheet.

#### 2.5.4 Statistical Analysis Conducted for Experimentation

GraphPad Prism 9 software was selected for statistical analysis. All data are shown as mean ± standard error of the mean (SE). For two groups, an unpaired two‐tailed *t*‐test was performed for intergroup comparisons. In the comparison of the three groups, we employed a one‐way analysis of variance (ANOVA). We established statistical significance with a *p* value of less than 0.05 (*p* < 0.05).

## 3 Results

### 3.1 Genetic Causality and Correlation Between Interleukin‐6 Receptor Subunit Beta and AD

#### 3.1.1 Instrumental Variable Selection for Interleukin‐6 Receptor Subunit Beta Levels

**FIGURE 2:**
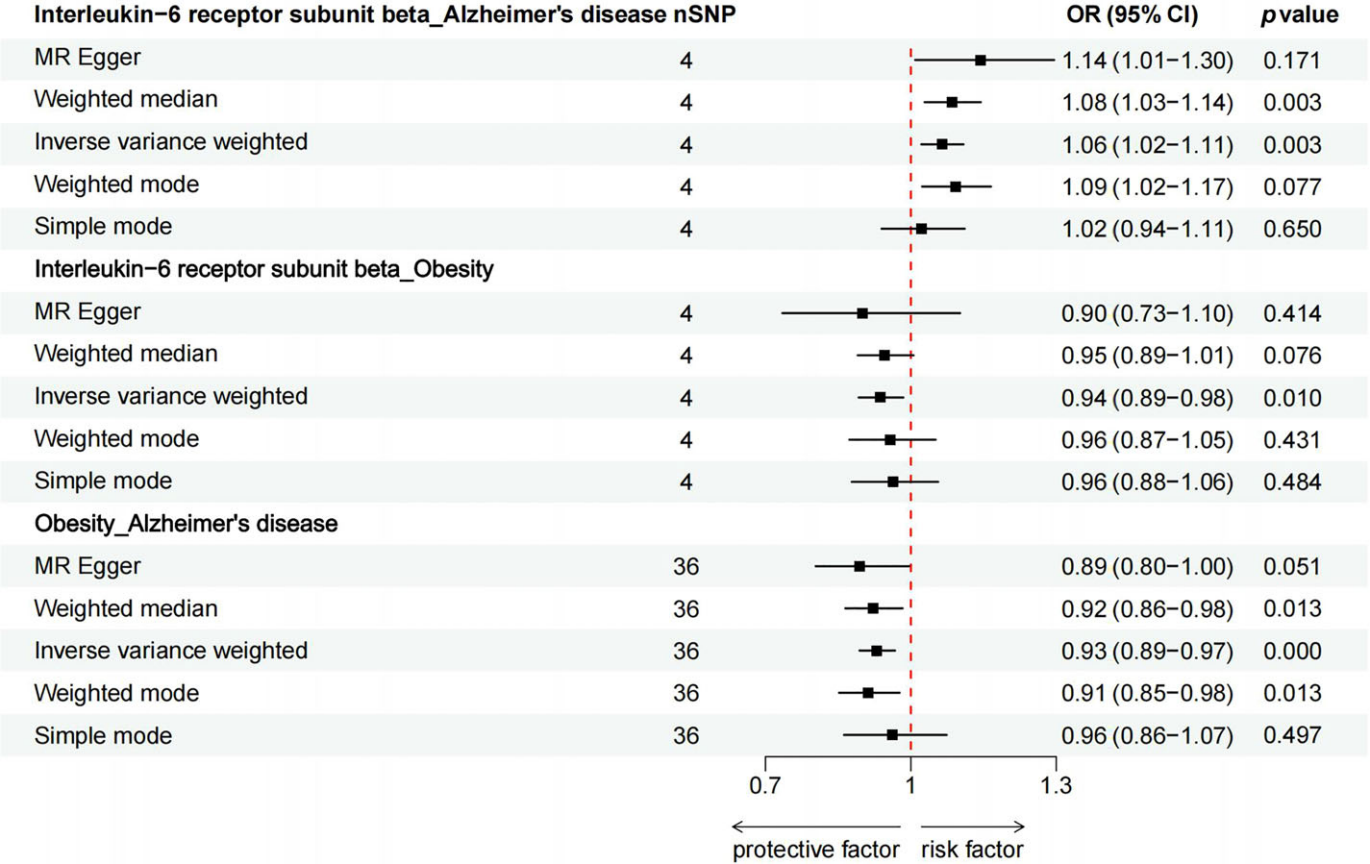
Forest map visualizes the causal link among interleukin‐6 receptor subunit beta, obesity, and AD.

Figure 2 visualizes the causal link among Interleukin‐6 receptor subunit beta, obesity, and AD using a forest map. The genetic determinants of Interleukin‐6 receptor subunit beta levels are seen in Figure 2, which ultimately included four SNPs (rs11574765, rs11927405, rs3862628, and rs635634) as instrumental variables (IVs). These genetic determinants all exceeded the genome‐wide significance threshold (*p* < 5 × 10^−8^) and showed strong tool strength (*F* statistic > 10). The specific SNP information can be found in Table S1.

#### 3.1.2 MR Analysis: The Role of Interleukin‐6 Receptor Subunit Beta in AD

**FIGURE 3:**
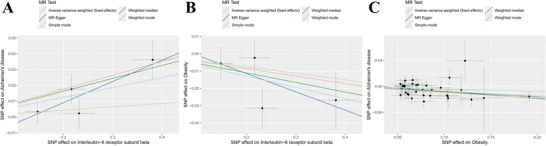
Scatter plot illustrating MR analysis for the interleukin‐6 receptor subunit beta, obesity, and the causality of AD. (A) The scatter plot demonstrates the causal connection between the Interleukin‐6 receptor subunit beta and AD. (B) The scatter plot highlights the causal link between the Interleukin‐6 receptor subunit beta and obesity. (C) The scatter plot depicts the causal connection between obesity and AD.

The IVW method served as the primary analytical approach to explore the possible causal link between the Interleukin‐6 receptor subunit beta and AD. The analysis indicated a significant causal relationship, with an odds ratio (OR) of 1.064 (95% Confidence Interval [CI]:1.021‐1.109,*p* = 0.003). The weighted median method corroborated these findings, yielding an OR of 1.085 (95% CI: 1.028–1.144,*p* = 0.003), consistent with the main IVW results. Furthermore, MR Egger regression analysis suggested that directed pleiotropy is unlikely to skew the results (intercept = −0.018; *p* = 0.356). Notably, MR‐Egger regression also provided an OR of 1.064 (95% CI: 1.009–1.296, *p* = 0.171), aligning with earlier findings. Given that the weighted median method offers greater accuracy than the MR‐Egger approach, it supports the hypothesis that higher levels of Interleukin‐6 receptor subunit beta elevate the risk of AD. Figure 2 illustrates the results of Mendelian randomization of Interleukin‐6 receptor subunit beta levels and AD. The scatter plot illustrating the MR analysis results is available in Figure 3.

#### 3.1.3 Verification of Mendelian Randomization Hypothesis

**FIGURE 4:**
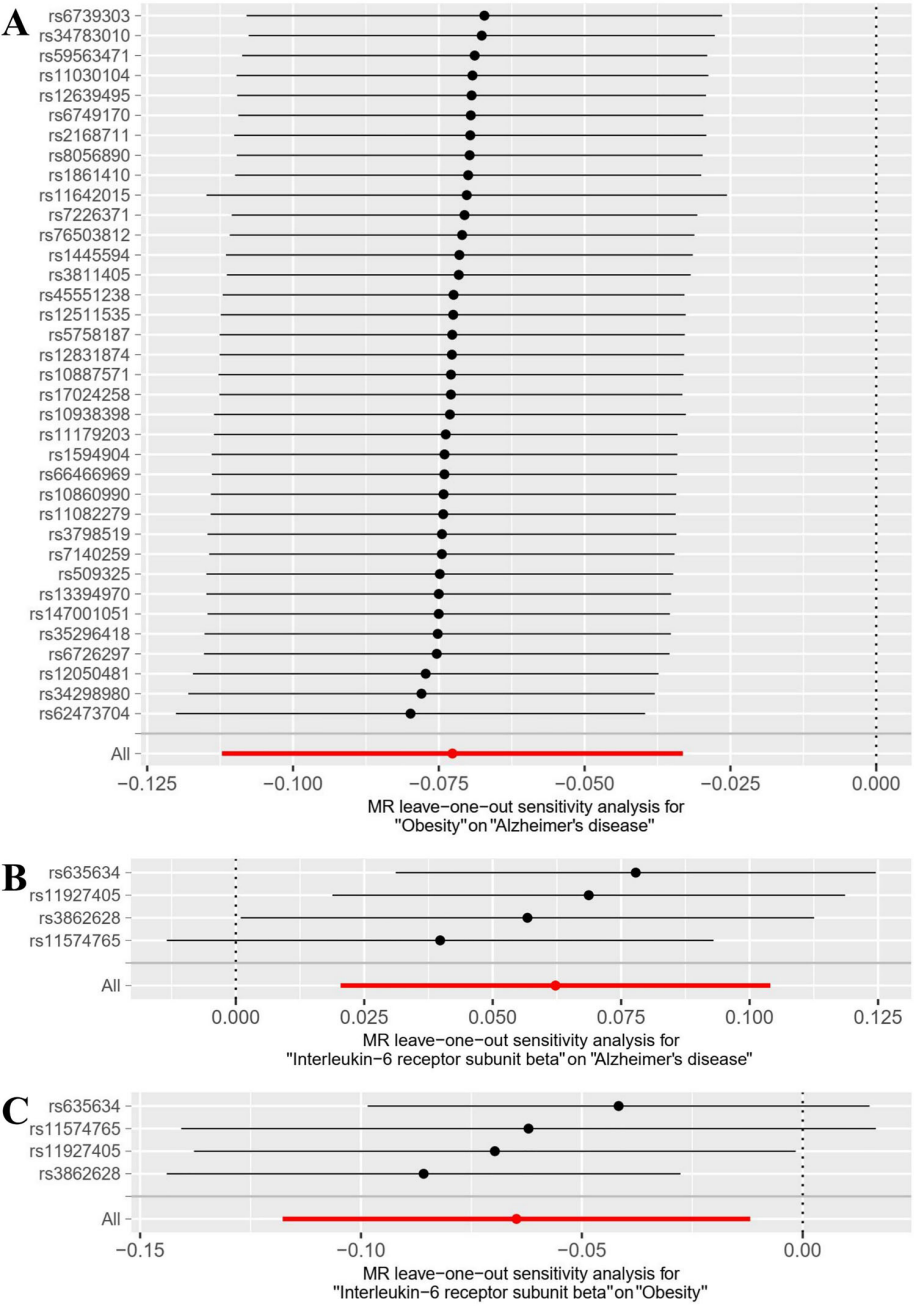
LOO analysis diagram to verify the causal link between interleukin‐6 receptor subunit beta, obesity, and AD. (A)The results of LOO analysis chart to verify the causal connect between Interleukin‐6 receptor subunit beta and AD. (B) The results of LOO analysis chart to verify the causal link between Interleukin‐6 receptor subunit beta and obesity. (C) The results of LOO analysis chart to verify the causal link between obesity and AD.

The selection process for SNPs adheres to a rigorous genome‐wide significance threshold (*p* < 5×10^−8^). This benchmark ensures the validity of the second MR hypothesis by minimizing directed pleiotropy (*p* = 0.356). Heterogeneity tests within the MR framework showed no significant variability (*p* = 0.426). MR‐PRESSO did not reveal any potential horizontal pleiotropy (*p* = 0.427). We also conducted LOO analysis to assess each SNP's impact on the causal estimate. After each SNP was removed individually, MR analysis was performed on the remaining SNPs. The results show that the addition of each SNP makes a significant contribution to establishing causality. Our comprehensive verification of the core principles of the MR approach ensures that the relationship between the analyzed Interleukin‐6 receptor subunit beta levels and AD is not affected by unknown confounders or intermediate variables. The above LOO analysis results are shown in Figure 4.

### 3.2 Genetic Causality and Correlation Between Interleukin‐6 Receptor Subunit Beta and Obesity

#### 3.2.1 Instrumental Variable Selection for Interleukin‐6 Receptor Subunit Beta Levels

We identified four genome‐wide significant SNPs for use as IVs. These genetic determinants meet the criteria for genome‐wide significance (*p* < 5 × 10^−8^), with an F statistic much greater than 10. The specific SNP information can be found in Table S3.

#### 3.2.2 MR Analysis: The Role of Interleukin‐6 Receptor Subunit Beta in Obesity

We employed IVW methods, MR‐Egger regression, and weighted median analysis to assess the causal influence of interleukin‐6 receptor subunit beta levels on obesity. The IVW analysis revealed that elevated levels of Interleukin‐6 receptor subunit beta slightly decreased the risk of obesity (IVW, OR = 0.9372, 95% CI: 0.8921–0.9847, *p* = 0.010). The weighted median approach yielded similar findings (OR = 0.9462, 95% CI: 0.8903–1.0057, *p* = 0.076), while the MR‐Egger method indicated a non‐significant result (OR = 0.8997, 95% CI: 0.7347–1.1018, *p* = 0.414). Figure 2 illustrates the results of Mendelian randomization of Interleukin‐6 receptor subunit beta levels and obesity. The scatter plot depicting the effects of these SNPs on individuals with obesity is presented in Figure 3.

#### 3.2.3 Verification of Mendelian Randomization Hypothesis

We conducted a sensitivity analysis of MR Studies on the causal link between interleukin‐6 receptor subunit beta and obesity. Using Cochran's Q test (*p* = 0.204), no evidence of heterogeneity in causality was found for these SNPs. Additionally, the MR‐PRESSO test did not reveal any potential horizontal pleiotropy (*p* = 0.204). We also conducted LOO analysis to assess the influence of each SNP on the overall causal estimate. These findings imply that all SNPs significantly contribute to establishing causality. The above LOO analysis results are shown in Figure 4.

### 3.3 Genetic Causality and Correlation Between Obesity and AD

#### 3.3.1 Selection of Obesity Instrumental Variables

After removing palindromes and fuzzy SNPs, we retained 36 genome‐wide important SNPs for use as IVs. These genetic markers satisfy the criteria for genome‐wide significance(*p* < 5 × 10^−8^) and exhibit an *F* statistic significantly exceeding 10. The specific SNP information can be found in Table S3.

#### 3.3.2 MR Analysis: The Role of Obesity in AD

We used IVW method, MR‐Egger regression, and weighted median analysis to explore the causal relationship between obesity and AD. IVW analysis showed that increased obesity moderately reduced the risk of AD (IVW, OR = 0.9299, 95% CI: 0.8939–0.9674, *p* ＜0.001). The weighted median approach (OR = 0.9220, 95% CI: 0.8646–0.9832, p = 0.033) corroborated these findings. Similarly, the MR‐Egger method (OR = 0.8949, 95% CI: 0.8035–0.9966, *p* = 0.051) aligned with these results. Figure 2 illustrates the results of Mendelian randomization of obesity and AD. The corresponding scatter plot of the effects of these SNPs on AD individuals is shown in Figure 3.

#### 3.3.3 Verification of Mendelian Randomization Hypothesis

We conducted a sensitivity analysis of the causal link between obesity and AD. Using the Cochran Q test (*p* = 0.780), no evidence of causal heterogeneity was found for these SNPs. Potential horizontal pleiotropy was not detected in the MR‐PRESSO test (*p* = 0.708). In addition, we used LOO analysis to assess the influence of each SNP on the overall causal estimate. These results indicate that all SNPs play a crucial role in establishing causality. The outcomes of the LOO analysis are depicted in Figure 4.

### 3.4 Experimental Verification

#### 3.4.1 Levels of Interleukin‐6 Receptor Subunit Beta (gp130), IL‐6, and OSM in Serum and Hippocampal Tissue of Mice Across Groups

**FIGURE 5:**
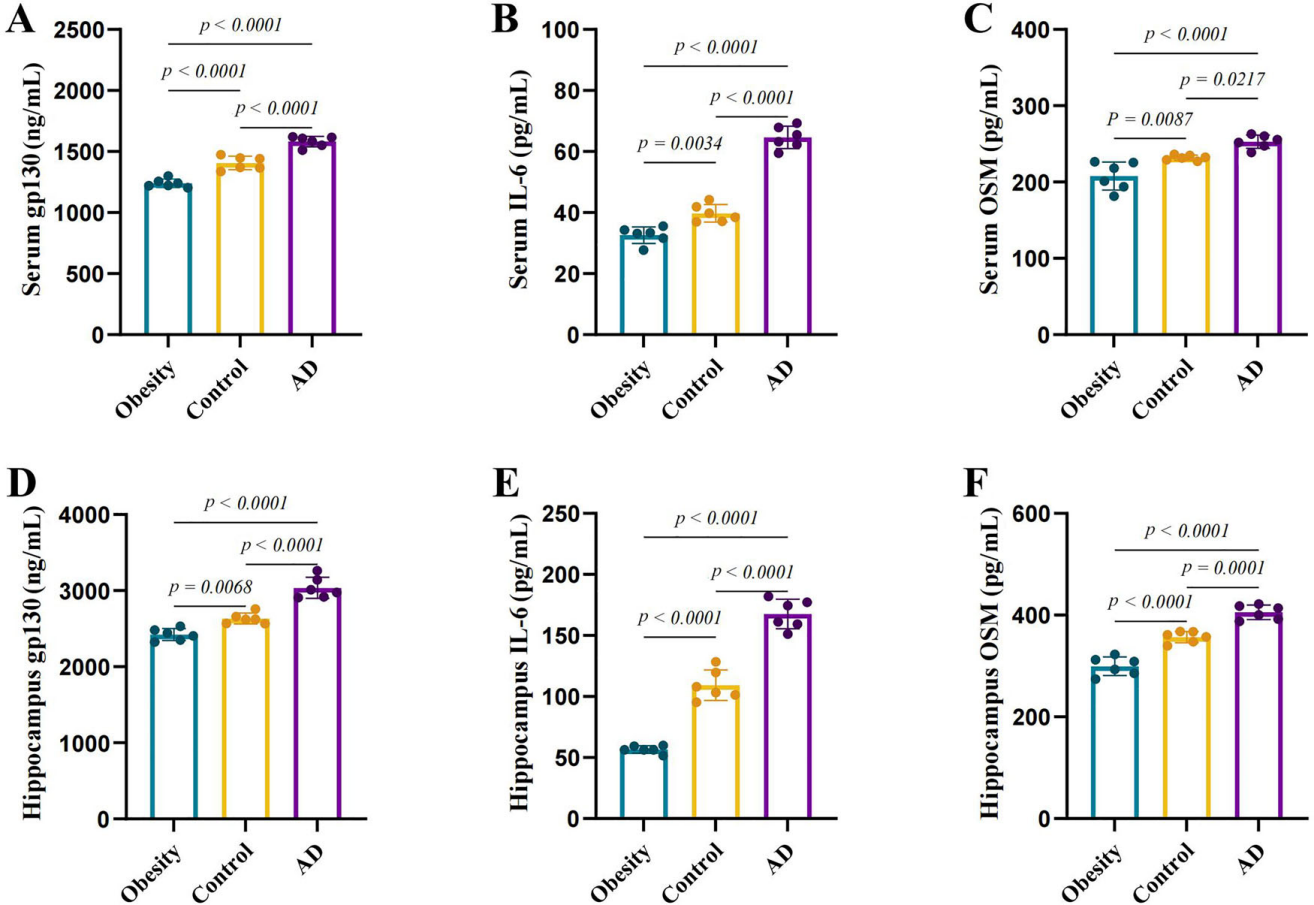
The variations in interleukin‐6 receptor subunit beta, IL‐6, and OSM levels in serum and hippocampal tissue among the mouse groups are examined. (A) Serum concentrations of interleukin‐6 receptor subunit beta across each group of mice. (B) Serum concentrations of IL‐6 in all mouse groups. (C) Serum concentrations of OSM in each group of mice. (D) Interleukin‐6 receptor subunit beta levels in the hippocampal tissue of mice from each group. (E) IL‐6 levels in the hippocampal tissue of mice in every group. (F) OSM levels in the hippocampal tissue of mice in each respective group.

In order to explore whether the development of obesity and AD are related to interleukin‐6 receptor subunit beta and IL‐6 cytokine family expression levels, we employed ELISA to quantify the levels of interleukin‐6 receptor subunit beta, IL‐6, and OSM in serum and hippocampal tissue from the control group, obesity group, and AD group. The findings revealed that, in comparison to the control group, the levels of Interleukin‐6 receptor subunit beta, IL‐6, and OSM in the serum and hippocampal tissue of mice in the obesity group were significantly reduced (*p* < 0.01). In contrast, the levels of these markers in the serum and hippocampus of mice in the AD group were significantly elevated (*p* < 0.05 or *p* < 0.01). Furthermore, the Interleukin‐6 receptor subunit beta, IL‐6, and OSM levels in the serum and hippocampal tissue of the AD group surpassed those in the obesity group (*p* < 0.01).The ELISA results are depicted in Figure 5.

#### 3.4.2 Pearson Correlation Analysis of Interleukin‐6 Receptor Subunit Beta (gp130) in Mice Serum and Hippocampus

**FIGURE 6:**
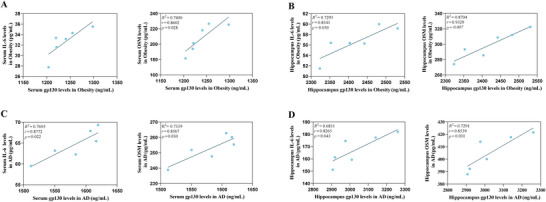
Correlation of IL‐6, OSM and interleukin‐6 receptor subunit beta in serum and hippocampal tissue of the obesity group and AD group mice. (A) Correlation between IL‐6, OSM, and interleukin‐6 receptor subunit beta levels in the serum of obesity mice (*p*  =  0.046,  *r * =  0.8186, 95% CI: 0.0017–0.1305 and *p*  =  0.028, *r*  =  0.8602, 95% CI: 0.0835–0.8589). (B) Correlation between IL‐6, OSM, and interleukin‐6 receptor subunit beta levels in the hippocampal tissue of obesity mice (*p*  =  0.030, *r*  =  0.8541, 95% CI: 0.0050–0.0598 and *p*  =  0.007, *r*  =  0.9329, 95% CI: 0.1006–0.3329). (C) Correlation between IL‐6, OSM, and interleukin‐6 receptor subunit beta levels in the serum of AD mice (*p*  =  0.022, *r*  =  0.8722, 95% CI: 0.0181–0.1327 and *p*  =  0.030, *r*  =  0.8567, 95% CI: 0.0288–0.3219).(D) Correlation between IL‐6, OSM, and interleukin‐6 receptor subunit beta levels in the hippocampal tissue of AD mice (*p*  =  0.043, *r*  =  0.8265, 95% CI: 0.0134–0.1603 and *p*  =  0.031, *r*  =  0.8539, 95% CI: 0.1006–0.3329).

Pearson's correlation showed that IL‐6 and OSM concentrations in serum and hippocampal tissue of the obesity group mice were positively correlated with interleukin‐6 receptor subunit beta concentrations. Similarly, the levels of IL‐6, OSM and interleukin‐6 receptor subunit beta in serum and hippocampus of AD group mice were also positively correlated. Figure 6 displays the results of the Pearson correlation analysis.

## 4 Discussion

We performed a comprehensive MR analysis and validated it with animal experiments. Our study suggests a potential association among Interleukin‐6 receptor subunit beta, obesity, and AD, with genetically predicted obesity possibly serving as a mediating factor in this relationship. Additionally, the accumulation of interleukin‐6 receptor subunit beta may be linked to reduced obesity and could contribute to an increased risk of AD. Further exploration of the relationships among these three factors may provide new insights and targets for clinical interventions in obesity and AD.

Interleukin‐6 receptor subunit beta, or gp130, conveys signals from the IL‐6 cytokine family, which includes OSM, CNTF, IL‐6, and IL‐11 (Jones et al. 2011). This subunit plays a crucial role in both classical and trans‐signaling pathways of IL‐6. In trans‐signaling, IL‐6 receptor (IL‐6R) is released from the cell surface, resulting in a soluble form of IL‐6R (sIL‐6R). The complex formed by IL‐6 and sIL‐6R enhances the expression of interleukin‐6 receptor subunit beta and promotes pro‐inflammatory responses (Rose‐John et al. 2023). Studies have shown that the trans‐signaling of IL‐6 can stimulate the transcription of amyloid precursor proteins and participate in neurodegenerative diseases such as AD (Rothaug et al. 2016). Our MR analysis indicates a positive association between the interleukin‐6 receptor subunit beta and AD, suggesting that the interleukin‐6 receptor subunit beta may increase the risk of AD at the genetic level. Consistently, our animal experiments demonstrate that, compared to the control group, the levels of gp130, IL‐6, and OSM are elevated in the serum and hippocampus of the AD group, indicating that increased levels of interleukin‐6 receptor subunit beta may influence the development of AD. However, these findings require confirmation through further research and exploration of their potential biological mechanisms.

The interleukin‐6 receptor subunit beta may have a negative causal effect on obesity. A previous study showed that administering CNTF cytokines to both genetically obese mice and diet‐induced obese mice resulted in weight loss, while CNTF variants with impaired interaction between Interleukin‐6 receptor subunit beta and leukemia inhibitory factor receptor (LIFR) lacked activity, suggesting that CNTF has an anti‐obesity effect, which may be mediated through the activation of gp130 and LIFR (Gloaguen et al. 1997). Similarly, OSM exerts biological effects by binding to a heterodimer complex composed of OSM receptor β subunit and interleukin‐6 receptor subunit beta. After OSM treatment in diet‐induced obese mice, lipid contents in the fat, liver, and serum decreased and total lipid and triacylglycerol contents in feces increased. This indicates that OSM may effectively lower body fat content by diminishing lipid intake, thereby playing a therapeutic role in obesity management (Komori et al. 2015). All the above studies suggest that the interleukin‐6 receptor subunit beta may play a role in influencing obesity. Our MR results indicate that interleukin‐6 receptor subunit beta may be a protective factor against obesity. Subsequent animal experiments show that, compared to normal mice, the levels of gp130, IL‐6, and OSM in the hippocampus and serum of obese mice are reduced, which is consistent with some studies, suggesting that an increase in gp130 may help reduce obesity, but more in‐depth research is needed to confirm.

Previous studies have shown that a higher BMI later in life, especially obesity later in life, reduces the risk of AD. A longitudinal study conducted in the United States and Canada revealed that AD patients who maintained a higher BMI exhibited increased brain volume and superior cognitive function relative to those with normal weight. Notably, cognitive decline in obese individuals progressed more slowly than in their normal‐weight peers during follow‐up, suggesting a potential protective effect of obesity against old‐age AD (Sun et al. 2020). Another prospective cohort study involving almost exclusively Asians similarly showed a causal effect between late‐life obesity and AD risk, with a lower risk of AD in those with a higher BMI and a lower risk in those with a slower rate of BMI decline (Hughes et al. 2009). In addition, a study utilizing a TaF34‐AD rat model found that diet‐induced obesity via a HCHF diet during the early stages of Alzheimer’s disease progression yielded beneficial outcomes. Obesity at this early phase was shown to increase myelination, which may thereby help prevent or slow cognitive decline and reduce the risk of AD (Ahmed et al. 2024). In our MR analysis, we found a negative causal effect between obesity and AD, which provides some evidence that obesity may reduce the risk of AD. In summary, the interleukin‐6 receptor subunit beta may reduce obesity and may thereby increase the risk of AD. Further research is needed to elucidate the potential mechanisms behind the causal relationships among the interleukin‐6 receptor subunit beta, obesity, and AD.

Our study is the first to employ the MR method to explore the causal relationship among interleukin‐6 receptor subunit beta, obesity, and AD, arriving at credible conclusions. We derived credible conclusions using a robust GWAS database with a sufficient sample size to validate our findings. The MR method we chose avoids the interference of positional confounders and improves the accuracy of the analysis results through multiple sensitivity analyses. In addition, we also used animal experiments to verify the MR analysis results.

However, it is vital to acknowledge certain limitations. First of all, the GWAS database we selected was mostly from the European population and could not represent the global population. Second, the number of interleukin‐6 receptor subunit beta SNPs meeting the inclusion criteria is small, and obtaining more interleukin‐6 receptor subunit beta SNPs meeting the inclusion criteria will make the analysis results more convincing. Finally, the data we used did not capture the age of the population and could not prove whether obesity in middle age or old age reduced the risk of AD.

In summary, this pioneering study investigates the causal link among interleukin‐6 receptor subunit beta, obesity, and AD, providing corresponding ideas and evidence for clinical prevention and treatment of AD.

## Author Contributions

**Yu Liu**: visualization, writing – original draft, validation. **Nan Song**: conceptualization, methodology, writing – review and editing, visualization. **Qun Wang**: software, data curation, investigation, visualization. **Peng Cui**: conceptualization, supervision, writing – review and editing. **Dongyu Min**: conceptualization, funding acquisition, writing – review and editing, supervision.

## Conflicts of Interest

The authors declare no conflicts of interest.

## Peer Review

The peer review history for this article is available at https://publons.com/publon/10.1002/brb3.70772

## Supporting information




**Supplementary Tables**: brb370772‐sup‐0001‐tableS1‐S3.xlsx

## Data Availability

The data that support the findings of this study are available from the corresponding author upon reasonable request.
